# Light-Induced Antioxidant Phenolic Changes among the Sprouts of Lentil Cultivar

**DOI:** 10.3390/antiox13040399

**Published:** 2024-03-27

**Authors:** You Rang Park, Soon-Jae Kwon, Ji Hye Kim, Shucheng Duan, Seok Hyun Eom

**Affiliations:** 1 Graduate School of Green-Bio Science, College of Life Sciences, Kyung Hee University, Yongin 17104, Republic of Korea; yrspark@khu.ac.kr (Y.R.P.); jhkim96@khu.ac.kr (J.H.K.); dsc97@khu.ac.kr (S.D.); 2 Advanced Radiation Technology Institute, Korea Atomic Energy Research Institute, Jeongeup 56212, Republic of Korea; soonjaekwon@kaeri.re.kr; 3 Smart Farm Science, College of Life Sciences, Kyung Hee University, Yongin 17104, Republic of Korea

**Keywords:** lentil sprout, LED, UV, phenolic compounds, HPLC-ESI/Q-TOF MS, antioxidant activity

## Abstract

Lentil is a leguminous crop with a high content of health-beneficial polyphenols. Lentil sprouts are popularly consumed in fresh vegetable markets, although their phytochemical qualities are not well understood. In this study, we investigated the accumulation of phenolics in lentil sprouts in response to photosynthetic and stress light qualities, including fluorescent light (FL), red LED (RL), blue LED (BL), ultraviolet A (UV-A), and ultraviolet B (UV-B). Three lentil cultivars, Lentil Green (LG), French Green (FG), and Lentil Red (LR), were used to evaluate sprouts grown under each light condition. The adequate light intensities for enhancing the antioxidant activity of lentil sprouts were found to be 11 W/m^2^ under photosynthetic lights (FL, RL, BL), and 1 W/m^2^ under stress lights (UV-A, UV-B). Subsequently, HPLC-ESI/Q-TOF MS analysis was conducted for the quantitative analysis of the individual phenolics that were accumulated in response to light quality. Four main phenolic compounds were identified: ferulic acid, tricetin, luteolin, and kaempferol. Notably, tricetin accumulation was significantly enhanced under BL across all three lentil cultivars examined. Furthermore, the study revealed that the other phenolic compounds were highly dependent on FL, BL, or UV-B exposure, exhibiting cultivar-specific variations. Additionally, the antioxidant activities of lentil extracts indicated that BL was most effective for LG and FG cultivars, whereas FL was most effective for enhancing antioxidant activity of LR cultivars as the sprouts grew.

## 1. Introduction

Lentils (*Lens culinaris*) are legumes known for their rich content of plant-based proteins and dietary fibers, along with a high concentration of essential micronutrients, including vitamins and minerals, making them a valuable addition to a balanced diet [1]. Compared to other legumes, lentils have higher levels of polyphenols, saponins, and phytosterols, which offer various potential health benefits, such as antioxidant, anti-inflammatory, antihyperglycemic, and anticancer effects [2,3,4]. Notably, lentils contain significant amounts of polyphenols such as phenolic acids and flavonoids, which contribute substantially to their antioxidant activities. It has also been suggested that a diet rich in lentils can prevent aging and cardiovascular diseases [5].

The germination of legume seeds, including lentils, enhances the synthesis of health-beneficial bioactive compounds, making sprouts nutritionally superior to seeds [6]. This increase in nutritional value is attributed to its higher polyphenol content, including flavonoids, which enhance the antioxidant properties of sprouted seeds [7,8]. Additionally, lentil sprouts exhibit a lower starch content and reduced levels of antinutrients such as tannins and phytic acid, contributing to their increased preference and consumption. Sprouted lentils and their flour are utilized in a variety of foods like bread, soup, and salads, serving as ingredients for health promotion [9,10].

With the expansion of controlled environmental systems for plant cultivation, artificial lighting has become an essential component of greenhouses and plant factories. Microgreens, sprouts, and baby leafy vegetables are particularly well suited for cultivation in artificially controlled agricultural facilities [11]. Light absorption during the early plant growth stages crucially influences growth and metabolism. Red and blue light spectra affect the metabolic processes related to the activity of primary and secondary metabolites, which are essential for plant growth functions, such as chlorophyll synthesis, stomatal opening, enzyme production, photosynthesis, and stem elongation [12]. Red light influences seed germination via phytochrome receptors [13], whereas blue light enhances root growth, leaf expansion, and phenol content [14,15]. Studies have shown that UV light, including UV-A and UV-B, positively affects the accumulation of antioxidants and enzyme activity, highlighting the potential of light-controlled cultivation to produce vegetables with higher contents of beneficial compounds [16,17].

Numerous studies have investigated the efficacy of single light sources in enhancing functional compounds in sprout crops. However, the impact of light quality on the accumulation of functional compounds during the germination of lentil seeds and sprouts remains unclear. Efficient light control is crucial for producing healthy sprouts in artificial lighting facilities. Therefore, the aim of this study was to investigate the effects of FL, RL, BL, UV-A, and UV-B on phenolic accumulation during a 6-day germination process in three lentil cultivars and their correlation with antioxidant activity.

## 2. Materials and Methods

### 2.1. Seed Materials and Sprout Cultivation

Lentil cultivars used in this research were LG, FG, and LR, which were purchased from Asia Seed (Seoul, Republic of Korea). Seeds were soaked in tap water for 2 h. After removing seed coats, thirty seeds were placed in a plant culture dish (ø 90 mm × h 40 mm) containing four layers of sterilized gauze with 3 mL of distilled water. The seeds were grown for 6 days under different light conditions in a cultivation room with constant temperature and humidity at 26 ± 2 °C and 70 ± 5%, respectively. At 2, 4, and 6 days after germination (DAGs), ten individuals were collected from each dish for a total of four dishes per treatment. All of the lentil seeds and sprouts, including shoot, root, and cotyledon, were lyophilized at −80 °C in a freeze dryer (IlshinBioBase, Dong-ducheon, Republic of Korea).

### 2.2. Light Conditions

Lentil sprouts were grown under five individual light sources, including FL (λmax = 437, 543, 610 nm, Philips Co., Hamburg, Germany), LED RL (λmax = 654 nm, D9RBN10SC, Plant Husbandry, Suwon, Republic of Korea), LED BL (λmax = 448 nm, D9RBN10SC, Plant Husbandry, Suwon, Korea), UV-A (λmax = 374 nm, F71T12 100W, Philips Co., Hamburg, Germany), and UV-B (λmax = 317 nm, Narrowband TL 20W/01—RSUlterviolet-B, Philips Co.). The light spectrum emitted by the lamps utilized in this experiment is depicted in Figure S1. Light intensity was regulated by adjusting the distance between the sprout and the light source, using a photo-radiometer (HD 2302.0, Delta OHMSRL, Marconi, Italy). To evaluate the impact of light intensities on sprout quality, photosynthetic lights (FL, BL, and RL) were set at 25, 50, 100, and 200 μmol·m^−2^·s^−1^, respectively; meanwhile, UV light (UV-A and UV-B) intensities were adjusted to 0.5, 1, and 2 W·m^−2^, accordingly. The conversion from μmol·m^−2^·s^−1^ to W·m^−2^ was performed using the approximation method described in a previous study [18]. Seeds were subjected to continuous irradiation with various light qualities throughout the entire cultivation period. Given that the germination process for legumes typically occurs under dark conditions, and to more thoroughly investigate the influence of light quality on the accumulation of functional components in the sprouts, a continuous dark treatment was employed as a control group.

### 2.3. Extraction Method for Analysis

The lyophilized lentil samples were ground to a fine powder using a stainless-steel ball (ø 9.52 mm) with vortex mixer. Sample extraction was conducted following the methods previously reported in our study [19], with some modification. Briefly, one hundred milligrams of the sample were mixed with 1 mL of 80% aqueous methanol. The mixtures were sonicated for 2 h and incubated for 12 h in a 22 °C shaking incubator (Daewon Science Inc., Bucheon, Republic of Korea). The mixtures were centrifuged at 12,800× *g* at 24 °C for 10 min. The supernatant was collected and used for the analysis of total phenolic content (TPC), total flavonoid content (TFC), and antioxidant activities.

### 2.4. TPC and TFC Analysis

TPC and TFC of lentil sprouts were measured using a previously published method [20]. TPC and TFC were expressed as milligrams of gallic acid and catechin equivalents per gram of dry weight (DW), respectively.

### 2.5. Acid Hydrolysis and HPLC Analysis of Phenolic Compounds

Acid hydrolysis of the lentil samples was performed for the HPLC analysis of flavonoid aglycones according to a previous study, with minor modifications [19]. Briefly, 30 mg of lentil samples were mixed with 1 mL of 1.2 M HCl diluted with 50% aqueous methanol, and incubated at 90 °C for 2 h. Then, the mixtures were cooled in ice and were centrifuged at 12,800× *g* for 5 min. The supernatant was collected and filtered using a 0.45 μm PTFE filter (Futecs Co., Ltd., Daejeon, Republic of Korea) for HPLC analysis. Individual phenolic compounds analysis was performed with reversed-phase HPLC (Waters 2695 Alliance HPLC, Waters Inc., Milford, MA, USA) with an octadecylsilane column (Prontosil 120-5-C18 SH (5 μm, 250 × 4.6 mm), Bischoff, Leonberg, Germany). The gradient elution of mobile phases (0.1% formic acid in water and 0.1% formic acid in acetonitrile) followed the previously described method [19]. The injection volume was 10 μL. The flow rate was maintained at 1 mL/min. Peaks were detected at 360 nm using a Waters 996 photodiode array detector (Waters Inc.). Individual phenolics in lentil samples were quantified with kaempferol standard (Sigma-Aldrich Co., St. Louis, MO, USA).

### 2.6. HPLC-ESI/Q-TOF MS Analysis of Phenolic Compounds

The LC instrument was an Ultimate3000 (Thermo Scientific, Waltham, MA, USA) equipped with a Waters Cortex T3 (2.1 mm × 150 mm, 1.6 μm). The mobile phases were 0.1% formic acid in water (Solvent A) and 0.1% formic acid in acetonitrile (Solvent B). The total elution time for the analysis was 60 min at a flow rate of 0.25 mL/min. The mobile phases were conducted using the following gradient: 0–0.5 min, 3% (B); 15 min, 15% (B); 50 min, 100% (B); 55 min, 100% (B); 55.1–60 min, 3% (B). The operating temperature was maintained at 45 °C. The MS spectrometer used for the mass analysis was a Triple TOF 5600+ (AB Sciex, Redwood City, CA, USA) equipped with electrospray ionization (ESI). The analysis was performed in positive and negative modes. The MS and MS/MS scan ranges were set from *m*/*z* 100 to 2000 and *m*/*z* 30 to 2000, respectively. The collision energy of the positive ion mode was 10 eV and that of the negative ion mode was –10 eV. The electrospray source parameters such as curtain gas, collision gas, ion spray voltage, and source temperature were 25 psi, N2, 4.5 kV, and 500 °C, respectively. Interpretation of the targeted data was performed using Element-viewer software (version 2.1.1, viewer 20181214). The identification of phenolic compound peaks in lentil sprouts was performed by matching the MS results with those reported in previous studies [21,22,23].

### 2.7. Antioxidant Activities

The antioxidant activities were determined by the 2,2′-azino-bis (3-ethylbenzothiazoline-6-sulphonic acid) (ABTS) and 2,2-diphenyl-1-picrylhydrazyl (DPPH) (Sigma Aldrich Co., St Louis, MO, USA) radical scavenging activities using the previously published method [24]. The extracts from 2.3 extraction methods were used for these assays. The activities were expressed as milligrams of vitamin C equivalents (VCEs) per gram of DW.

### 2.8. Statistical Analysis

All experimental measurements were performed in triplicate. SAS software (Enterprise Guide 7.1 version; SAS Institute Inc., Cary, NC, USA) was used for statistical analysis. One-way analysis of variance (ANOVA) was used to test for differences in the mean values between different samples. Significant differences were indicated at a level of *p* < 0.05 by Duncan’s multiple range test. The correlation between the phenolic content and antioxidant activity was determined using Pearson’s correlation coefficient.

## 3. Results and Discussion

### 3.1. Determination of Light Intensities on Sprout Qualities

The cultivar of LG was used to ascertain the effective light intensity for enhancing sprout growth, TPC, and antioxidant activity in lentil sprouts across various light qualities. The changes of growth lengths, TPC, and ABTS radical scavenging activity in lentil sprouts on 6 DAGs across varying light qualities and intensities, are depicted in Table 1. Among photosynthetic light treatments, shoot lengths decreased with increased intensity beyond 11 W/m^2^. In terms of root lengths, FL and RL demonstrated the highest values at 5.5 W/m^2^, whereas BL reached its peak at 11 W/m^2^. Under UV light conditions, both shoot and root lengths tended to decrease with higher intensity. TPC and antioxidant activity levels in sprouts on 6 DAGs showed overall similar patterns. TPC levels in FL remained consistent across all intensity levels, whereas RL and BL demonstrated intensity-dependent variability. RL exhibited the highest TPC levels at 11 W/m^2^, and BL reached its peak values at 11 and 22 W/m^2^. Regarding UV lighting, while TPC under UV-A exhibited no notable differences across intensities, comparable TPC levels under UV-B were noted at 0.5 and 1 W/m^2^, though the content diminished at 2 W/m^2^. ABTS radical scavenging activity peaked in FL and RL at 11 W/m^2^, whereas BL displayed its highest values at 11 and 22 W/m^2^. Analogous to the TPC findings, UV-A demonstrated no significant variations in antioxidant activity across intensity levels, whereas UV-B manifested the highest levels at 1 W/m^2^.

**Figure 1:**
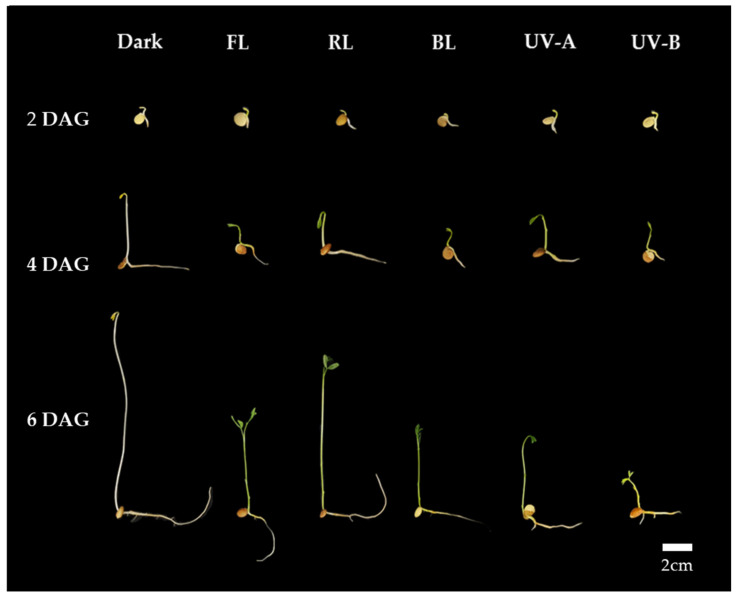
Morphology of lentil sprouts of LG grown under dark, fluorescent light (FL), red LED (RL), blue LED (BL), ultraviolet A (UV-A), and ultraviolet B (UV-B) for 2, 4, and 6 days after germination (DAGs).

Thus, it is suggested that a uniform optimal light intensity exists for enhancing shoot length growth, TPC, and antioxidant activity in lentil sprouts under each light quality. As per the TPC and antioxidant activity results for lentil sprouts on 6 DAGs, the highest levels were commonly observed at an intensity of 11 W/m^2^ in the FL, RL, and BL. Furthermore, the optimal intensity for shoot length growth in the shoots of the sprouts was determined to be 11 W/m^2^. Although UV-A demonstrated no significant differences in TPC and antioxidant activity according to intensity, higher intensity levels were found to adversely affect growth. UV-B was observed to inhibit both growth and the synthesis of substances at intensity levels above 1 W/m^2^. With an increase in UV light intensity, sprout growth becomes inhibited, whereas antioxidant activity experiences an enhancement. Consequently, applying an intensity of 11 W/m^2^ in the photosynthetic lights and 1 W/m^2^ in the UV lights is deemed most appropriate for lentil sprouts. Based on the above results, we applied the appropriate intensity of each light to investigate the impact of light quality on antioxidant phenolics across various lentil cultivars. The typical morphology of LG lentil sprouts, which were irradiated with various light qualities at the determined intensities for 2, 4, and 6 days, is shown in Figure 1.

### 3.2. Comparison of Phenolic Content in Different Lentil Cultivars in Response to Light Qualities

#### 3.2.1. TPC and TFC Changes of Lentil Sprouts

**Figure 2:**
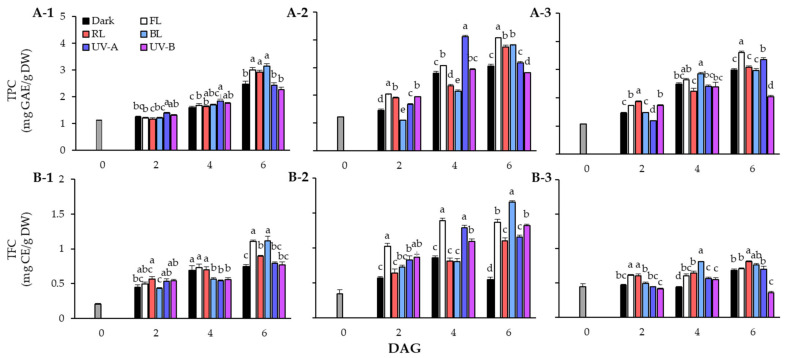
Changes of total phenolic content (TPC) (**A**) and total flavonoid content (TFC) (**B**) in lentil sprouts of LG, FG, and LR after 6 days after germination (DAGs). The numbers (1 to 3) beside the capital letters (A and B) indicate LG, FG, and LR, respectively. The values at 0 DAGs indicate the contents of seeds. Values represent means with standard error of three replicates. The letters (a–e) in each column indicate significant differences at *p* < 0.05 by Duncan’s multiple range test.

The TPC of lentil seeds and sprouts in response to light quality during the germination period is shown in Figure 2A. The TPC of lentil sprouts increased following the germination stage, and the TPC content was higher in sprouts than in seeds of all three cultivars. The TPC of the seeds was 1.12 mg GAE/g DW in LG, 1.23 mg GAE/g DW in FG, and 1.06 mg GAE/g DW in LR. In the case of LG, the TPC showed a clear distinction at 6 DAGs, with significantly higher accumulation under FL, RL, and BL conditions than under dark and UV light conditions. In particular, the sprouts treated with BL exhibited over a 27% increase in phenolic content compared with those grown under dark conditions. In terms of FG, high levels of TPC were found in the FL treated group in sprouts at 2 DAGs and 6 DAGs. Interestingly, in sprouts at 4 DAGs, the accumulation of phenolics due to UV-A was found to increase significantly but decreased at 6 DAGs. In the case of LR, the TPC of sprouts grown under UV-A continued to increase, showing a higher content than that under RL and BL at 6 DAGs. However, TPC of sprouts on 6 DAGs was found to be at the highest level under FL.

In TFC in response to light quality, the contents of LG sprouts showed an increasing trend in all experimental groups at the germination stage. Although TFC steadily increased under FL, RL, and BL conditions, it did not increase significantly under UV light. The sprouts on 6 DAGs showed particularly high TFC under FL (1.10 mg CE/g DW) and BL (1.11 mg CE/g DW). The FG cultivar had high sprout TFC under FL conditions from the beginning of germination. The sprouts grown under FL (1.02 mg CE/g DW) exhibited TFC increase of more than 2.9 times compared to the seeds (0.35 mg CE/g DW) on 2 DAGs, and were 1.8 times higher than those grown in dark conditions. However, on 6 DAGs, TFC increased significantly under BL (1.66 mg CE/g DW) rather than FL (1.37 mg CE/g DW). Additionally, the FG sprouts showed higher flavonoid accumulation under UV light than under dark conditions. In the case of LR, TFC did not significantly increase following the germination stage compared with the other two cultivars. However, the photosynthetic lights resulted in a higher flavonoid concentration compared to UV light, within TFC of LR sprouts.

The results of this study showed that the phenolic content of sprouts from three lentil cultivars was effectively enhanced when irradiated with either FL or BL. According to Świeca et al. [25], it was found that the phenolic acid and flavonoid content increased with continuous white LED irradiation during the lentil germination period. Meanwhile, for 6 DAGs sprouts, FL and BL were confirmed to be light factors that increased the total phenolic content of lentil sprouts. The TPC and TFC of LG cultivar, as well as the TFC of FG, demonstrate that BL is more effective at enhancing phenolic compound accumulation than RL. This finding aligns with the observation from our previous study indicating that BL exposure leads to increased flavonoid accumulation during the germination stage of buckwheat sprouts [26]. According to studies using soybean and pea sprouts, BL irradiation promoted the accumulation of TPC during seed germination, and in the case of soybeans, it showed a better effect than green LED [27,28]. Thus, the effectiveness of FL in increasing the phenolic content of lentil sprouts may be attributed to the inclusion of BL wavelengths. During germination, three lentil varieties exhibited distinct accumulation patterns of phenolic compounds. FG demonstrated the most significant increases in total TPC and TFC under light treatment compared to other cultivars. The rates of increase in TPC for LG and LR were similar, whereas the enhancement of TFC in LG surpassed that in LR (Figure 2). Ghasemzadeh et al. [29] explored the impact of light on TPC and TFC variations in young ginger varieties, indicating that accumulation significantly varied with cultivars, light exposure, and plant parts. Bravi et al. [30] analyzed the effect of sprouting on phenolics in five *Camelina sativa* (L.) Crantz cultivars, revealing that the germination process significantly enhanced TPC in seeds, with variations in the extent of enhancement across cultivars. It is widely acknowledged that phenolics biosynthesis commences with the shikimic acid pathway, progressing to the phenylpropanoid pathways to synthesize phenolic acids, hydroxycinnamic acids, flavonoids, and more [31,32]. The accumulation process of phenolics is markedly influenced by factors including gene expression levels, enzyme activities, and the availability of required substrates in plants. These factors likely contribute to the varied accumulation patterns of TPC and TFC observed among different lentil varieties during germination. Light quality serves as a crucial environmental factor, stimulating enzyme activities within the phenylpropanoid pathways during seed germination and thereby enhancing the synthesis of phenolic compounds [33,34]. This provides an explanation for the variation in phenolics in lentil sprouts under different light conditions.

#### 3.2.2. Individual Phenolic Compound Changes in Lentil Sprouts

##### Identification of Phenolic Compounds in Lentil Seeds and Sprouts

**Figure 3:**
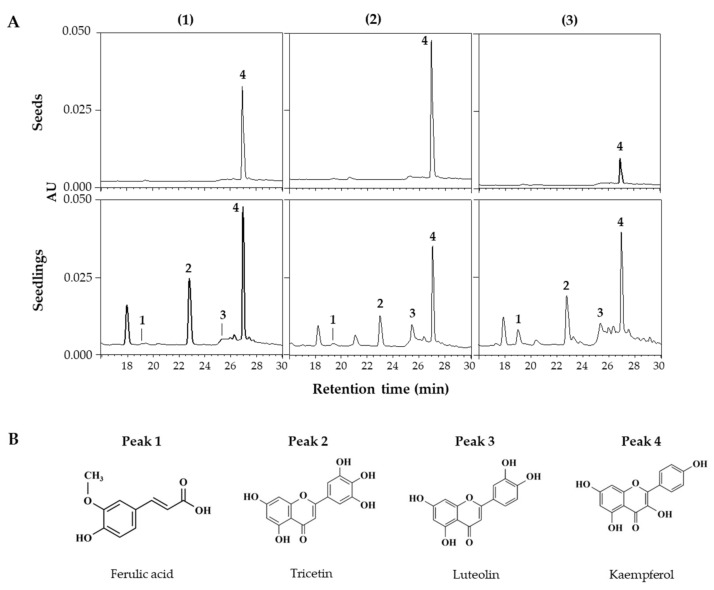
HPLC chromatograms of phenolic compounds in seed and seedlings of LG (**1**), FG (**2**), and LR (**3**) grown for 6 days under FL (**A**). Chemical structures of phenolic compounds detected from lentil extracts (**B**). The chromatograms were detected at 360 nm. Peak numbers in chromatograms correspond to the peak number of Table 2.

HPLC and HPLC-ESI/Q-TOF MS analyses were performed to identify and quantify changes in individual phenolic compounds within lentil extracts. Figure 3A shows the HPLC chromatograms of extracts from seeds and 6 DAGs sprouts grown under FL across the three lentil cultivars. This chromatogram showed peaks detected from the seeds and newly appearing peaks from the sprouts, with variations in the detected peaks among the cultivars, indicating differences in the synthesized compounds. HPLC-ESI/Q-TOF MS analysis identified the corresponding substances from the four major peaks in the HPLC chromatogram (Figure 3B; Table 2), with peaks 1–4 being ferulic acid, tricetin, luteolin, and kaempferol, respectively.

##### Individual Phenolic Compound Variation in Lentils during Germination

**Figure 4:**
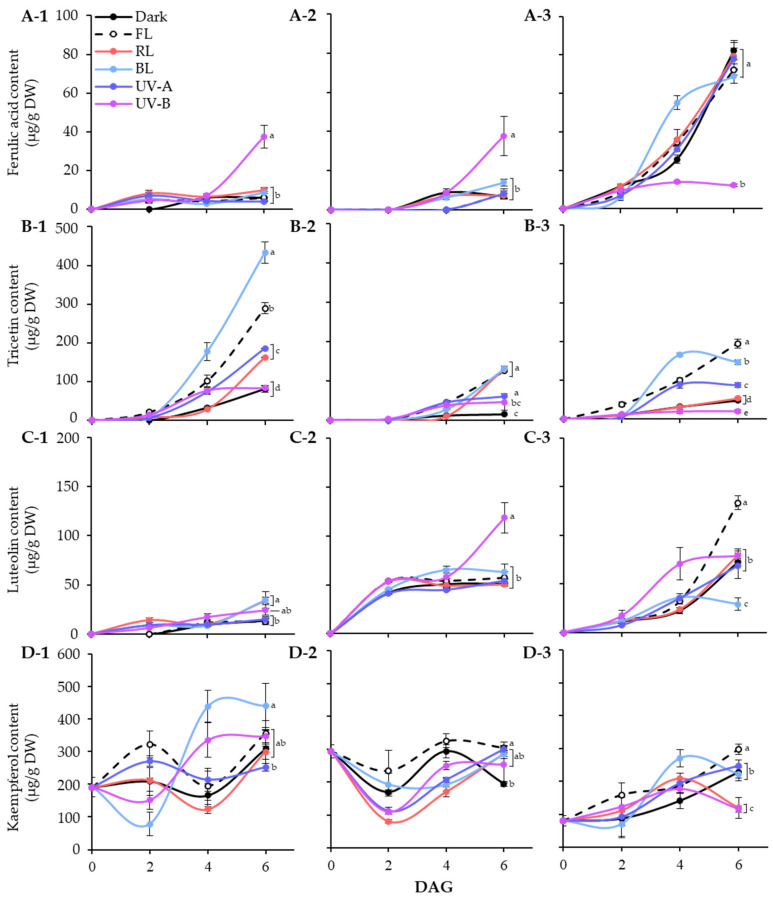
Quantification of major phenolic compounds, ferulic acid (**A**), tricetin (**B**), luteolin (**C**), and kaempferol (**D**), in lentil sprouts of LG, FG, and LR. The numbers (1 to 3) beside the capital letters (A to D) indicate LG, FG, and LR, respectively. Significant differences comparing the phenolic content of lentil sprouts within each day after germination were indicated with the letters (a–e) at *p* < 0.05 according to Duncan’s multiple range test. DAGs: days after germination.

In lentil seeds, only kaempferol was detected, regardless of cultivars. After germination, light induced different accumulation patterns of phenolic substances in different varieties of lentils. Figure 4 showed the accumulation patterns of phenolic compounds in lentil sprouts grown under different light qualities during 6 days of germination.

In LG, ferulic acid was detected in light treated sprouts at 2 DAGs. The level was specifically increased at 6 DAGs under UV-B exposure, whereas it kept in lower levels under other conditions. Light quality showed distinct effects on tricetin and kaempferol accumulation, especially BL for 6 days significantly increased the contents, as shown in Figure 4(B-1,D-1). Meanwhile, the effects of light quality on luteolin accumulation were minimal. At 6 DAGs, the UV-B and BL treatment groups showed relatively high levels compared to other light and dark treatments. Thus, tricetin and kaempferol accumulation in LG sprouts increased under the influence of BL during germination, whereas only ferulic acid responded to UV-B exposure.

The FG cultivar also exhibited specific light quality responses with respect to accumulation of phenolics. The ferulic acid and luteolin content in FG sprouts significantly increased under UV-B exposure at 6 DAGs. Light treatment also enhanced the increase in tricetin content. At 6 DAGs, tricetin contents were higher in sprouts exposed to FL, RL, and BL than the control and UV light, with similar levels across photosynthetic light qualities. The kaempferol content in FG sprouts decreased early in germination under all light conditions and then increased to a level comparable to that of seeds by 6 DAGs, except under dark conditions. Therefore, in FG sprouts, the accumulation of ferulic acid and luteolin by UV-B occurred actively, whereas the accumulation of tricetin and kaempferol by photosynthetic lights occurred relatively effectively.

Unlike the other two cultivars, the ferulic acid in the 6 DAGs LR sprouts was at least five times higher under non-UV-B light conditions than UV-B treated sprouts. The fact that there was no significant difference in ferulic acid content under all conditions except UV-B indicates that the accumulation of ferulic acid was inhibited by UV-B irradiation in LR. Tricetin and kaempferol showed similar accumulation patterns, with BL leading up to 4 DAGs. By 6 DAGs, the increase in those under BL was less significant compared with other light conditions, especially FL, which showed a higher accumulation effect. Moreover, FL significantly increased luteolin content in LR sprouts, with 1.8 times more content than under dark conditions by 6 DAGs. Accordingly, the content of all four phenolics was enhanced under FL conditions during LR sprout cultivation.

Overall, the germination process increased phenolic content in lentil sprouts, varying across cultivars. Dark conditions induced the highest accumulation of ferulic acid in LR sprouts at 6 DAGs, reaching 82.47 μg/g DW (Figure 4A). The pattern of ferulic acid accumulation induced by light quality appears to be specifically influenced by UV-B exposure. Similar patterns were observed in LG and FG sprouts, where UV-B exposure specifically enhanced their content, with 37.56 μg/g DW in LG and 37.73 μg/g DW in FG, compared to other light conditions. Conversely, LR exhibited a reverse pattern, where UV-B significantly reduced ferulic acid content, displaying 12.23 μg/g DW at 6 DAGs. Wang et al. [35] demonstrated that UV-B irradiation significantly increased the content of phenolics, including ferulic acid, in barley seedlings. Moreira-Rodríguez et al. [36] found that a low-dose UV-B treatment for 2 h led to an increase in ferulic acid content in broccoli sprouts compared to 8-day-old controls, whereas a 24 h exposure had a minimal effect. However, sprouts subjected to high-dose UV-B treatment exhibited a tendency towards reduced ferulic content. The impact of lentil varieties on the accumulation of flavonoids under different light qualities also revealed diverse patterns (Figure 4B–D). BL and FL lights significantly facilitated the accumulation of tricetin across the three varieties, with LG exhibiting the highest increase, while FG and LR showed relatively lower amounts. UV-B and FL significantly promoted luteolin accumulation in FG and LR, respectively, whereas LG exhibited insensitivity to light, resulting in a gradual increase in luteolin content throughout germination. BL promoted the accumulation of kaempferol in LG and mitigated the reduction trend of its content in FG and LR observed other different light conditions. The impacts of BL and UV-B on phenolic accumulation in plants, including sprouts, are well-documented [37,38]; however, the influence of light qualities on phenolic substances in various lentil varieties remains less understood. Our results highlight the distinct patterns of phenolics accumulation induced by different light qualities across lentil varieties. These differences may be caused by different light signal perception ability and metabolic enzyme activities of lentil cultivars [39,40].

### 3.3. Antioxidant Activities of Lentil Seeds and Sprouts

**Figure 5:**
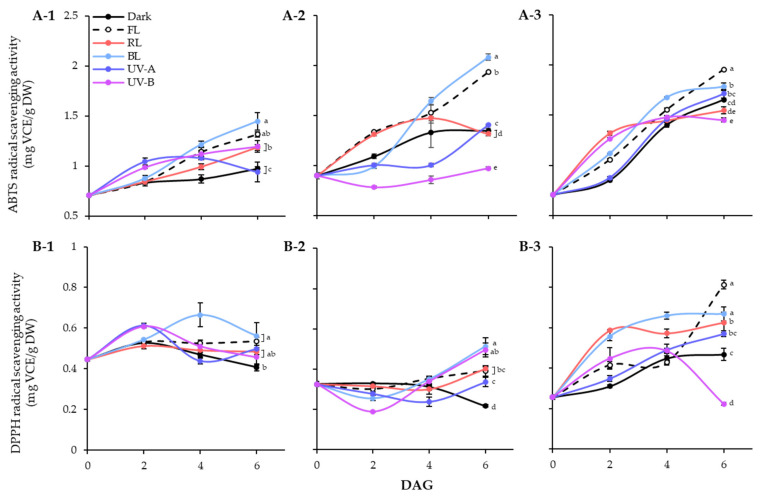
ABTS (**A**) and DPPH (**B**) radical scavenging activities of lentil sprouts of LG, FG, and LR. The numbers (1 to 3) beside the capital letters (A and B) indicate LG, FG, and LR, respectively. Values represent means with standard error of three replicates. Significant differences comparing the antioxidant activities of lentil sprouts within each day after germination were indicated with the letters (a–e) at *p* < 0.05 according to Duncan’s multiple range test. DAGs: days after germination.

The effects of different light qualities on the antioxidant activities of the lentil extracts were demonstrated using ABTS and DPPH radical scavenging assays (Figure 5). In lentil seeds, the ABTS activities of LG, FG, and LR were 0.71 mg VCE/g DW, 0.90 mg VCE/g DW, and 0.70 mg VCE/g DW, respectively. The germination process gradually enhanced the ABTS radical scavenging abilities of lentil seed, regardless of light treated or not (Figure 5A). During 6 days germination under dark, LR showed the highest number of enhanced levels, followed by FG and LG. However, the effects of light qualities on ABTS radical scavenging ability were significantly differed on lentil cultivars. In the case of LG, all light treatment except UV-A increased the ABTS activities on 6 DAGs than dark. For LG and FG, only BL and FL dramatically enhanced the ABTS radical scavenging activity of sprouts. Overall, all three lentil cultivars showed high ABTS radical-scavenging activity under FL and BL conditions, with increasing antioxidant activity as germination progressed. Additionally, LG and FG showed more sensitive properties on BL and FL enhanced antioxidant activity than LR. The DPPH radical scavenging activity showed a different pattern from the ABTS activity of lentil sprouts (Figure 5B). The DPPH activities of seeds were 0.45 mg VCE/g DW, 0.33 mg VCE/g DW, and 0.26 mg VCE/g DW in LG, FG, and LR, respectively. The antioxidant activity of dark germinated sprouts was decreased in LG and FG, whereas increased in LR. Light treatments the enhanced the activity of each cultivar with different patterns. At 6 DAGs, LG and FG showed high levels of DPPH activity against BL, and LR showed high levels of DPPH activity against FL, which corresponded to the ABTS activity results.

Antioxidant activity was evaluated using ABTS and DPPH assays. Many studies have reported a strong positive correlation between phenolic compounds and ABTS and DPPH activities [41,42]. However, in this study, significant differences were found in the ABTS and DPPH activities. The ABTS method detects both hydrophilic and lipophilic antioxidants [43], whereas the DPPH method mainly detects hydrophobic antioxidants. Thus, the basis for the difference in activity between the two assays may be that antioxidant flavonoids in lentil seedlings exist mainly in the form of glycosides with hydrophilic complexes. Most flavonoids exist primarily as glycosides [44], supporting the inference that certain light-induced increases in flavonoid occur in the glycoside form. According to the results of this study, accumulation of phenolic compounds in lentil sprouts treated with FL or BL was involved in the high levels of ABTS-active substances measured. In contrast, the results of DPPH radical reaction analysis showed that lipophilic antioxidants that can be detected in lentil sprouts are present in small quantities in lentil sprouts. Therefore, the hydrophilic solvent extraction process will be helpful for developing and producing functional materials from lentils.

### 3.4. Correlation Analysis

The correlation between phenolic compounds and the antioxidant activities of lentil seeds and sprouts is shown in Table 3. The two antioxidant assays showed different trends in correlation with the quantitative changes in phenolic compounds in LG. The ABTS activity of LG extracts grown under different light conditions showed a strong positive correlation with phenolics (*p* < 0.001), except for ferulic acid. This is because ferulic acid accumulation is induced by UV-B irradiation, resulting in photoreaction accumulation that is different from that of other phenolics. Because the content of ferulic acid is lower than that of other phenolics, the total phenolic content and antioxidant activity may have minimal effects. Likewise, ferulic acid and luteolin contained in the FG extracts are phenolics that undergo an integrated reaction with UV-B and do not appear to have a significant correlation with other indicators. However, except for these specific cases, there was a significant positive correlation between the other phenolics and antioxidant activity.

## 4. Conclusions

In the present study, we investigated the influence of various light qualities on the accumulation of phenolic compounds in lentil sprouts during germination. Continuous exposure of all three lentil cultivars (LG, FG, and LR) to FL yielded sprouts with high phenolic compound contents after 6 days. Under BL conditions, LG and FG exhibited higher flavonoid content than LR. In the seeds of the three lentil varieties, only kaempferol was identified, while ferulic acid, tricetin, luteolin, and kaempferol constituted the predominant phenolic compounds in the lentil sprouts. These results demonstrate that germination boosts the metabolism of phenolic compounds in lentils. Further studies showed that BL played a significant role in the biosynthesis of tricetin and kaempferol during the germination of lentils. Concurrently, BL, FL, and UV-B were found to influence the accumulation of ferulic acid and luteolin in the lentil varieties to different extents. Additionally, germination markedly improved the antioxidant activity in lentil seeds. Under dark conditions, the LR variety exhibited the greatest increase in antioxidant activity, followed by FG and LG. BL treatment further elevated the antioxidant activity of lentil sprouts, displaying variable effects across the varieties. Specifically, FG and LG showed approximately double the increase in antioxidant activity, while LR experienced a relatively modest increase. Overall, while FL generally promotes phenolic accumulation across lentil cultivars, BL can enhance flavonoid content and antioxidant responses in certain cultivars. Although our findings suggest that certain light qualities are optimal for the accumulation of individual phenolics in different lentil cultivars, BL was suggested as the most effective for enhancing the nutritional and health benefits of lentil sprouts.

## Figures and Tables

**Table 1:** The growth lengths, TPC, and ABTS radical scavenging activity of LG sprouts after 6 DAGs under different quality and intensity of lights.

Light		Intensity (W/m^2^)	Shoot Length (mm)	Root Length (mm)	TPC (mg GAE/g DW)	ABTS Radical Scavenging Activity (mg VCE/g DW)
Dark			87.8 ± 4.17	65.1 ± 3.27	3.32 ± 0.04	1.63 ± 0.01
LED	FL	5.5	54.1 ± 1.28 b	73.2 ± 0.93 a	3.85 ± 0.06 a	1.64 ± 0.01 b
		11	60.0 ± 2.66 a	64.4 ± 2.06 b	3.74 ± 0.13 a	1.71 ± 0.01 a
		22	53.7 ± 1.99 b	59.8 ± 3.33 b	3.82 ± 0.09 a	1.67 ± 0.03 ab
		44	40.8 ± 1.12 c	36.2 ± 1.59 c	3.86 ± 0.10 a	1.68 ± 0.03 ab
	RL	5.5	64.6 ± 2.65 b	76.5 ± 3.76 a	3.63 ± 0.06 c	2.01 ± 0.04 c
		11	82.4 ± 2.56 a	52.7 ± 3.69 b	4.81 ± 0.09 a	2.35 ± 0.05 a
		22	67.3 ± 2.43 b	52.5 ± 1.78 b	3.77 ± 0.03 c	2.06 ± 0.02 bc
		44	80.8 ± 2.22 a	57.6 ± 3.03 b	4.12 ± 0.06 b	2.21 ± 0.05 ab
	BL	5.5	23.2 ± 1.29 c	17.6 ± 1.25 b	1.95 ± 0.02 c	1.38 ± 0.03 c
		11	69.4 ± 2.41 a	54.5 ± 4.88 a	4.22 ± 0.09 a	2.71 ± 0.06 a
		22	57.3 ± 1.11 b	19.3 ± 0.92 b	4.26 ± 0.07 a	2.61 ± 0.03 a
		44	24.8 ± 0.85 c	21.4 ± 2.07 b	2.98 ± 0.07 b	2.06 ± 0.04 b
UV	UV-A	0.5	58.7 ± 1.20 a	67.6 ± 3.21 a	3.59 ± 0.09 a	2.13 ± 0.02 a
		1	49.5 ± 0.70 b	63.3 ± 3.53 a	3.66 ± 0.09 a	2.18 ± 0.04 a
		2	44.3 ± 1.76 c	58.9 ± 1.63 a	3.91 ± 0.09 a	2.21 ± 0.04 a
	UV-B	0.5	17.9 ± 0.59 a	14.7 ± 1.25 a	3.30 ± 0.06 a	2.36 ± 0.03 ab
		1	13.3 ± 0.57 b	11.1 ± 0.48 b	3.13 ± 0.04 a	2.46 ± 0.06 a
		2	8.1 ± 0.46 c	9.6 ± 0.43 b	2.84 ± 0.04 b	2.20 ± 0.06 b

Values represent means with standard error of three replicates. Different letters (a–c) in each light quality of each column indicate significant differences at *p* < 0.05 by Duncan’s multiple range test.

**Table 2:** List of major phenolic compounds detected in the extracts from lentil seeds and sprouts.

Peak No.	*T_R_* (min)	UV λ_max_ (nm)	Formula	[M − H]^−^ (*m*/*z*)	Major Fragment Ions (*m*/*z*)	Identification
1	19.50	233, 277, 316	C_10_H_10_O_4_	193.0509	193.0494	Ferulic acid [21]
2	22.98	265, 362	C_15_H_10_O_7_	301.0362	301.0360	Tricetin [22]
3	25.44	252, 347	C_15_H_10_O_6_	285.0415	285.0416	Luteolin [23]
4	27.10	265, 365	C_15_H_10_O_6_	285.0412	285.0413	Kaempferol [23]

Peak numbers correspond to the peaks of chromatogram presented in Figure 2.

**Table 3:** Correlation coefficient analysis between phenolic compounds and antioxidant activities of lentil seeds and sprouts.

		TPC	TFC	Ferulic Acid	Tricetin	Luteolin	Kaempferol	ABTS	DPPH
LG	TPC	1							
	TFC	0.90771 ***	1						
	Ferulic acid	0.27315 ns	0.2853 ns	1					
	Tricetin	0.84568 ***	0.84259 ***	0.07838 ns	1				
	Luteolin	0.71894 ***	0.68607 ***	0.51332 ***	0.61685 ***	1			
	Kaempferol	0.54391 ***	0.5011 ***	0.3108 ***	0.63274 ***	0.52064 ***	1		
	ABTS	0.7372 ***	0.73592 ***	0.34687 ns	0.80862 ***	0.68328 ***	0.67223 ***	1	
	DPPH	−0.1735 ns	−0.05215 ns	−0.09125 ns	0.16968 ns	0.04678 ns	0.35085 **	0.28448 *	1
		TPC	TFC	Ferulic acid	Tricetin	Luteolin	Kaempferol	ABTS	DPPH
FG	TPC	1							
	TFC	0.72591 ***	1						
	Ferulic acid	0.29551 ns	0.39345 ns	1					
	Tricetin	0.79719 ***	0.72989 ***	0.37635 ***	1				
	Luteolin	0.16431 ns	0.3937 **	0.90713 ***	0.24307 ns	1			
	Kaempferol	0.58951 ***	0.65101 ***	0.4168 **	0.61618 ***	0.33536 ns	1		
	ABTS	0.46137 ***	0.42285 **	0.0998 ns	0.57938 ***	0.05519 ns	0.44922 ***	1	
	DPPH	0.38688 **	0.64315 ***	0.677 ***	0.64571 ***	0.64433 ***	0.597 ***	0.50947 ***	1
		TPC	TFC	Ferulic acid	Tricetin	Luteolin	Kaempferol	ABTS	DPPH
LR	TPC	1							
	TFC	0.73383 ***	1						
	Ferulic acid	0.89939 ***	0.80813 ***	1					
	Tricetin	0.765 ***	0.68743 ***	0.67382 ***	1				
	Luteolin	0.74408 ***	0.38578 **	0.62298 ***	0.49207 ***	1			
	Kaempferol	0.69067 ***	0.61567 ***	0.63232 ***	0.71488 ***	0.51268 ***	1		
	ABTS	0.93597 ***	0.65333 ***	0.79734 ***	0.76679 ***	0.7078 ***	0.69528 ***	1	
	DPPH	0.64679 ***	0.76752 ***	0.62988 ***	0.67849 ***	0.4016 **	0.59584 ***	0.65449 ***	1

Values marked with asterisks indicate significance by Pearson’s correlation analysis. *, **, and *** indicate *p* < 0.05, *p* < 0.01, and *p* < 0.001, respectively, also, ns indicates no significance. TPC, total phenolic content; TFC, total flavonoid content.

## Data Availability

Data are contained within the article and Supplementary Materials.

## Supplementary Materials

The following supporting information can be downloaded at: https://www.mdpi.com/article/10.3390/antiox13040399/s1, Figure S1: Light spectrum of the light sources, fluorescent light (A), red LED (B), blue LED (C), ultraviolet-A (D), and ultraviolet-B (E), used in this study.

## Abbreviations

LG	Lentil green
FG	French green
LR	Lentil red
TPC	Total phenolic content
TFC	Total flavonoid content
HPLC	High-performance liquid chromatography
FL	Fluorescent light
RL	Red LED
BL	Blue LED
UV-A	Ultraviolet-A
UV-B	Ultraviolet-B
