# Supplementary material for: Light-Induced Antioxidant Phenolic Changes among the Sprouts of Lentil Cultivar
Source: Antioxidants (Basel). 2024 Mar 27;13(4):399. doi: 10.3390/antiox13040399 (PMC11047427; doi:10.3390/antiox13040399)
Supplement: Supplementary file 1 [file antioxidants-13-00399-s001.zip › antioxidants-2932222-supplementary.pdf]

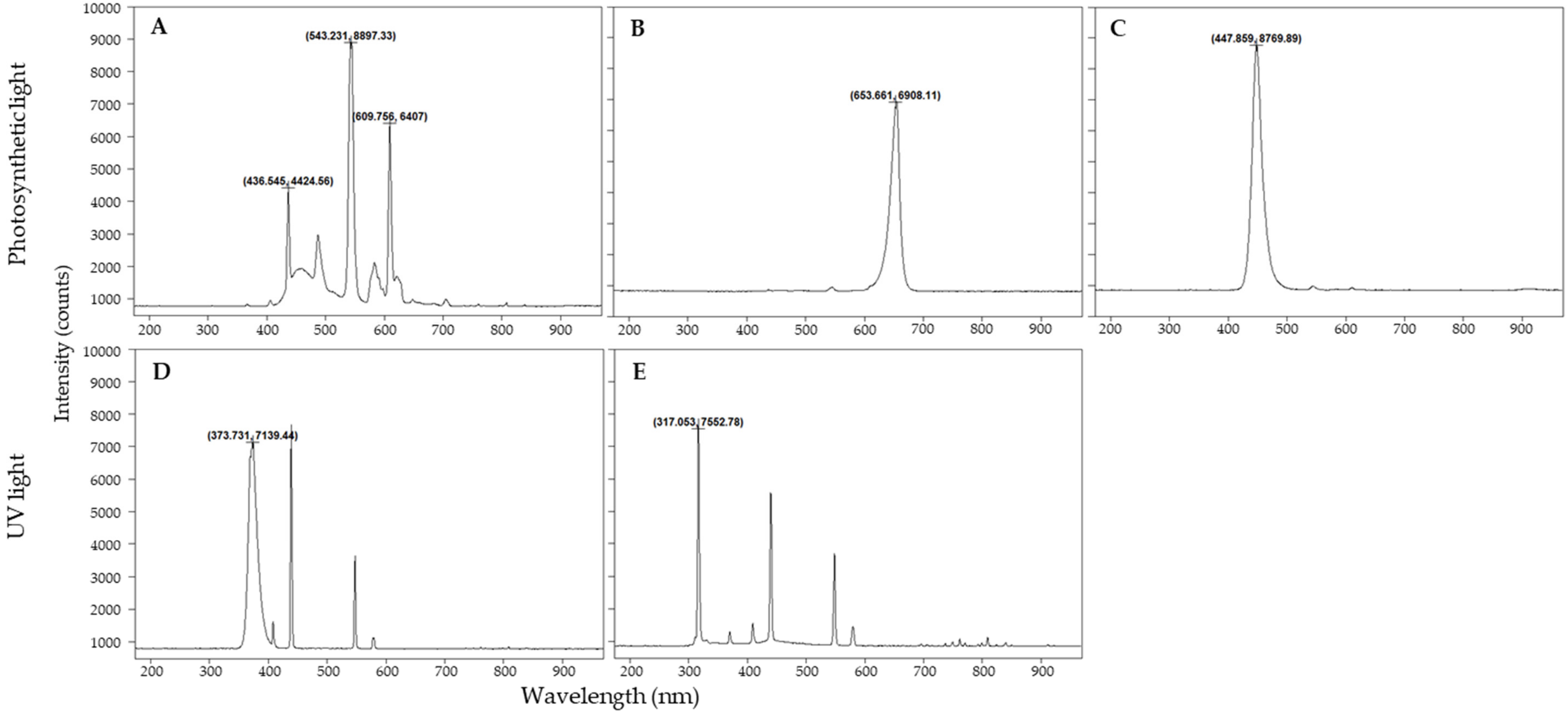

Supplementary Figure S1. Light spectrum of the light sources, fluorescent light (A), red LED (B), blue LED (C), ultraviolet-A (D), and ultraviolet-B (E), used in this study.
